# Safety climate in the Ghanaian printing industry

**DOI:** 10.1371/journal.pone.0278100

**Published:** 2022-11-28

**Authors:** Samuel Smith Esseh, Lucy Afeafa Ry-Kottoh, Mary Mawufemor Denyo

**Affiliations:** 1 Department of Publishing Studies, Kwame Nkrumah University of Science and Technology, Kumasi, Ghana; 2 Axiom Consult, Kumasi, Ghana; Ethiopian Public Health Institute, ETHIOPIA

## Abstract

The concept of safety climate has gained attention from safety experts as one of the most efficient and effective ways to deal with occupational accidents and injuries across industries. This paper explores the safety climate and the effect of employees’ demographic variables on the safety climate perception in the printing industry. We adopted the Safety Climate Scale (SCS) developed by Ghahramani and Khalkhali to measure the safety climate in the print manufacturing industry of Ghana. Our findings, based on all the dimensions in the scale, revealed an unsafe safety climate. Also, there was a correlation between demographic factors (age, gender, experience, and education) and perception of workplace safety climate. The major contribution of this paper is to extend empirical research that provides a greater understanding of the health and safety environment within the printing industry of Ghana and the personal and collective attitudes and patterns of behaviour that determine the commitment to organisations’ health and safety practices. These findings are important for managers in the printing industry because they provide evidence about the current safety climate so that management can take the action to reduce risks and improve performance. To improve the safety climate, we recommend that management and other stakeholders within the printing industry must commit and communicate effectively, embrace safety practices and procedures, and be more accountable and responsible to minimise the effects of a poor safety climate.

## Introduction

Safety climate, the perceived and assumed value placed on safety in an organisation has great potential to reduce workplace injury rates and positively influence safety performance in many business environments. Growing research supports the assertion that as an organisation’s safety climate improves, the organisation’s safety practices improve [1–3]. A safe business climate is, therefore, key in remediating work-related injuries, ill health and minimising work- hazards. There is growing empirical evidence, however, that suggests that occupational safety challenges are severe in the printing industry: from manual handling injuries, slip and trip injuries, to machinery injuries [4, 5]. The printing industry, classified as the sixth high-risk industry under the manufacturing industry, has over the years, witnessed serious accidents such as amputation, bruising and crushing caused by knives and clamps [6 (p8)]. Industry data showed that occupational exposure to printing processes, carbon black, printing inks and the nitro compound is possibly carcinogenic–leading to lung and bladder cancer–to humans [7]. In a related study, workers operating within the print production industry were found to be at high risk of contracting oesophageal, stomach, lung and ovarian cancers [8, 9].

Printing remains an important industry in the economy of Ghana. Although Ghana does not report a great deal of manufacturing output or employment statistics to international agencies [10], the last time an industrial census was conducted in 2003, about 26,000 manufacturing establishments were employing about 243,500 persons. As cited in Esseh, according to records from the Ministry of Trade and Industry dating back to 1983, the printing industry comprises 450 individual establishments that employed 13,173 people in mainly small and medium-sized companies [11]. The yearly value-added of the printing industry, together with other industries within the manufacturing sector, in 2016, was 5.63% of the US$55,010,000,000 GDP, which translates to US$3,097, 063,000 [12, 13]. The most common commercial printing types are: offset lithography, flexography and, more recently, a move into digital printing.

While there is a dearth of literature and empirical data on the state of safety climate of the printing industry in Ghana, the scarcely available literature shows that workers in the industry are exposed to a high level of injuries, diseases and risks (different occupational hazards) [14]. A series of unpublished research conducted by the Department of Publishing Studies, KNUST, (the only institution established by UNESCO and tasked with the responsibility of developing human resource capacity for the printing and publishing industry in Africa), found several undocumented occupational injuries and accidents in the printing houses within the Accra and Kumasi metropolis. Also, Addae et al. [15], in a study on the human and environmental impact of print production that included an analysis of the side effects of chemicals on press workers, showed work conditions exposed workers to asthma, lung diseases, respiratory diseases, irritations and constant headaches and catarrh. In another news article [16] and one other unpublished study by Esseh [11] several workers within the Ghanaian printing and other industries were exposed to several hazards: musculoskeletal disorders because of manual handling, slips and trips on production floors, high-temperature environment, and high noise and vibration [11]. Out of the total number of press houses in that study, 45% admitted that their workers have suffered an occupational hazard.

In a published empirical study on traumatic injuries among printing press workers in Ghana, Agbenorku et al. [14] found that press workers sustained many forms of injuries; laceration, fracture, dislocation, crushes, amputation and multiple fingernails torn off. Their study concluded that the prevalence rate of traumatic injuries among workers in the printing industry in Ghana was 67%. Boateng and Amedofu [17] also found that 7.9% of workers in the printing industry have evidence of noise-induced hearing loss (NIHL). Besides human suffering, loss of life, and environmental impact, the economic cost of industrial accidents on a nation cannot be trivialised. According to the International Labour Organization [18] (ILO), workplace injuries and illnesses result in about a 5% loss of Gross Domestic Product. Such a loss cost the Ghanaian economy an estimated $3.27 billion a year, given that Ghana’s current GDP stands at USD65.55 billion [13].

It is acknowledged that an individual’s attitudes, values, beliefs, risk perceptions and behaviours, called safety climate, are behind most occupational accidents. Employees’ actions or inactions based on their perceptions, values and beliefs about Safety Management Systems (SMS) will have a positive or negative influence on safety performance in any industrial setting [19]. Bigelow, as cited in Work [20] stated that “safety climate holds great potential in improving a company’s health and safety performance and reducing workplace injury rates” (para. 6). This safety climate is thus concomitant to the style and proficiency of an organisation’s health and safety management.

Although the economic and social significance of safety issues are apparent, and research on safety climate, particularly in the developed world, is growing, safety issues in the printing industry of Ghana have received only cursory attention from the scholarly community. Studies conducted have been ad hoc, and impressionistic rather than methodical. Little systematic and empirical research has scrutinised safety climate practices, and there continues to be a gap between practice and recommendations from the research. As Agbenorku et al. [14] and Puplampu and Quartey [21] observed, few empirical investigations have been conducted on Organisational Health Safety (OHS) within the printing industry of Ghana. A thorough review of the literature yielded only two pieces of empirical research within the meta-analysis on OHS. Undeniably, there are missing pieces in the literature [14, 21]; this calls for further empirical work to provide a greater understanding of the health and safety environment within the printing industry of Ghana.

Zohar [22] developed the concept of safety climate on the premise that employees’ perception of the organisational environment can be an immediate antecedent to behaviour. In the manufacturing industry, safety climate is described as the collective perception of workers about the value placed on safety by management as manifested by recent or current events. This is a demonstration of a safety culture in the behaviour and expressed attitude of employees [23]. The concept comes from a bottom-up perceptional approach that measures what workers think about safety culture in their company at a particular moment [24].

Thus, employees’ willingness to take practical action to deal with a problem is based on their perceptions of reality driven by the safety climate. The safety climate is, therefore, a reliable indicator among a variety of quantitative and qualitative data collection tools to predict workplace injuries [25]. A survey that asks employees how they, and their senior managers and immediate supervisors deal with safety issues, serves as a gauge and provides a focal point for changes to improve safety.

Safety climate is a multidimensional concept that includes several scales and a wide range of factors used to characterise individual perceptions of safety at a workplace [26–29]. The literature is replete with several developed safety climate dimensions. While previous studies attempt to explore safety climate factors and ways of improving and measuring them, there is a lack of consensus and little agreement on which dimensions or factors make up the most reliable [30–35]. As stated by Newaz [30 p739] and supported by other authors, “the lack of a unifying theoretical model in this area reflects the state of development of this field, where an inductive rather than a deductive approach is in operation” [36, 37]. Further, the lack of consensus could be attributed to the unique cultural differences among specific industries [38, 39] or that different instruments measure distinctly different safety climate concepts [26, 40].

The print manufacturing industry is one of the riskiest industries in terms of incident and accident rates [41, 42]. Although there is a general agreement to create a safety climate in the industries, few validated tools to measure important elements of a safety climate exist. In this study, we adopted the Safety Climate Scale for Manufacturing Industry (SCSMI) developed by Ghahramani and Khalkhali [43] to measure the safety climate in the print manufacturing industry of Ghana.

This paper examines the safety climate with particular emphasis on top management, supervisors and co-workers’ commitment to safety, which is relatively new and untested in the printing industry of Ghana. This research critically evaluates the safety environment within the industry through the application of a perception survey to understand the personal and collective attitudes and patterns of behaviour that define the commitment to an organisation’s health and safety practices. Broadly, the study centres on the overall effectiveness and safety practices as perceived by employees and how these practices define the industry’s safety climate.

Studies on how demographic factors impact risk perception have been carried out in multiple fields [44, 45], and considering the literature, demographic variables have significant roles to play regarding the perception of individuals on issues of safety climate [44]. Ameko [35] and Liao et al. [46] suggest that socio-demographic factors–gender, age, work experience, marital status, and educational attainment–influence work climate and may play a significant role in risk-taking behaviour at the workplace. These factors could contribute to human error, which was identified by Liao et al. [46] as a cause of construction accidents. Bayire [47] added that although a good safe work environment may lead to good behaviour and a dangerous work environment may lead to bad work behaviour, to a large extent individual characteristics hold sway in the way workers perceive risk in the workplace.

Ariss [48] indicated that the acquisition of knowledge, skills and employees’ unique abilities have the potential to empower workers to create a safe work environment. Thus, employees’ levels of education and gender influence their knowledge and ability to cope with work pressure. Women and men differ in their perceptions of risks [49] but males are more likely to behave in a risky way and get distracted when performing work [50–52].

Demographic/personal characteristics influence how workers interact with the safety climate in any organisation and, by extension, the rate at which injuries/accidents occur. However, demographic and other subgroup factors (e.g., employees’ educational background) and their effects on safety perceptions within the printing industry are under-researched. It is, therefore, necessary that empirical justification for using personal demographics as a validation technique is required if safety climate research is to progress.

This study, therefore, examined the following hypotheses:

H 1: There is a significant relationship between company size and employees’ perception of the safety climate.
H 2: There is a significant relationship between the safety climate perception of older employees and the safety climate perception of younger employees.
H 3: There is a significant relationship between gender and individual perception of the workplace safety climate.
H 4: There is a significant relationship between years of work experience and perception of the workplace environment.
H 5: There is a significant relationship between educational attainment and individual perception of the workplace climate.

## Materials and methods

Through a *perception survey*, this paper critically evaluates the safety environment within the printing industry of Ghana to understand and interpret personal and collective attitudes and patterns of behaviour that determine their commitment to an organisation’s health and safety practices. The population area for the study was the two most industrialised cities in Ghana: Accra in the Greater Accra Region, and Kumasi in the Ashanti Region. These two regions are the locations of roughly 90% of the total number of print production companies in Ghana. These printing companies create and manufacture products that communicate visually, including large format posters, newspapers, labels, books and other publications. They mostly employ the offset lithography printing processes; the most common method of printing used in Ghana.

Primary data was gathered from micro, small, medium and large-scale printing companies, from Accra and Kumasi. A forty-five-item Safety Climate Scale with a seven-dimension questionnaire: Safety Climate Scale for Manufacturing Industry (SCSMI), developed by Ghahramani and Khalkhali [43], was adopted and used in obtaining data from the population. Although the original items in the scale did not change, we added demographic variables to obtain data to test the hypotheses. The SCSMI comprises the following dimensions and the number of items included under each one:

1. Safety commitment and communication (16 items),
2. Safety involvement and training (8 items),
3. Positive safety practices (8 items),
4. Safety competency (3 items),
5. Safety procedures (4 items),
6. Accountability and responsibility (3 items),
7. Supportive environment (3 items).

The assessment of the major fit indices of the SCSMI shows the dimensional structure of the safety climate scale was satisfactory, and good data fit for the study. The reliability value for the 45-item scale was 0.96. This means that the survey measures the safety climate and predicts important outcomes, such as injuries. The internal consistency reliability of the safety climate components showed that the scale is appropriate for the current study.

We obtained permission from the owners of the presses, and respondents voluntarily gave consent to take part in the survey. Two hundred (200) questionnaires were administered to randomly selected workers drawn from the pre-press, press and post-press sections of the printing presses. 172 questionnaires were correctly completed and returned. A majority (76) representing 44.2% of the respondents, were from the press section.

The inclusion and exclusion criteria for the study were that, first, the organisations were registered members of the Ghana Printers and Paper Converters Association, the willingness of respondents to participate and the workers were full-time employees. Also, participants were drawn from Accra and Kumasi; two industrialised cities with the majority of printing companies. Other printing companies outside these two cities were excluded from the study.

We received written ethical approval from the Humanities and Social Sciences Research Committee of the Kwame Nkrumah University of Science and Technology, Kumasi Ghana, to carry out this research.

## Analysis of results

Mean and standard deviation, two basic statistical measurements were used to summarize the Likert-scale survey data. We introduce the Mean safety climate score (MSCS) which represents the average value on the Likert scale for all safety items to help evaluate the weak and strong perceptions regarding safety. The mathematical mean for the scale 1-2-3-4-5 is 3.0. So, in principle, results over 3.0 are positive or strong. Further, an independent t-test and analysis of variance (ANOVAs) were used to test the association between demographic characteristics and perceptions of safety climate, as measured by dimensions 1–7 in the SCSMI-45 survey.

Table 1 indicates the estimated power for a one-sample mean test. The estimated power assumed for the study is one (1) which is strong enough to reject the null hypotheses, assuming that the alternative hypotheses are true.

**Table 1:** Estimated power for a one-sample mean test t-test.

Estimated power for one sample mean test t-test	
alpha =	0.05
N =	172
delta =	0.7143
m0 =	2.5
ma =	3.0
sd =	0.7
Estimated power: power =	1

Table 2 shows the statistical analysis of the estimated sample size required to make the assumptions or hypotheses specified for the study. The mean for the null hypothesis (m0) is 2.5 and the mean for the alternative hypothesis (ma) is 3.0. The required estimated sample size is seventy-eight (78) and the change in value or delta is 0.7143 (that is the difference between the two means (m0) and (ma) /sd). This implies that in this study, approximately 0.7 or 70% of the variables accounted for the health and safety climate in selected printing firms in Ghana, leaving little difference unexplained.

**Table 2:** Estimated sample size.

Estimated Sample Size	
alpha =	0.05
power =	1
delta =	0.7143
m0 =	2.5
ma =	3
sd =	0.7
Estimated sample size: N =	78

## Findings and discussions

### Demographic analysis

Descriptive statistics were used to examine the demographic data using Statistical Package for the Social Sciences (SPSS) for Windows Version 18.0. The demographic factors include age, gender, education level, employment status and experience in the industry. The results are summarised in the tables below. Male employees formed a majority (82.6%) of the survey sample, indicating that the printing industry is a male-dominated one. As shown in Table 3 below, the age range of participants was between 18 and 60 years, with 49.4% of the participants between the ages of 18 and 30 years. The average age of the participants was 33, with a standard deviation of 9.45 (n = 172), showing a sizeable difference in age and pointing to the universality of the research conclusions. The respondents’ educational attainment concentrated between Senior High School (36%) and Bachelor/Diploma (31.9%). Respondents who had worked between 1 to 10 years accounted for more than half (68%) of the total participants in the study, with a few (16.3%) working for less than one year, and 15.1% for over 11 years.

**Table 3:** Demographics.

Demographic variance	No.	%	Demographic variance	No.	%
**Gender**			**Organizational size**		
Male	141	82	1–9	46	26.7
Female	31	18	10–29	49	28.5
			30–99	77	44.8
**Age**			**Departments**		
					
19–30	85	49.4	Administration	29	16.9
31–40	53	30.8	Prepress	26	15.1
41–50	19	11.0	Press	76	44.2
51–60	14	8.1	Post press	40	23.3
**Educational level**			**Position**		
					
No formal education	1	0.06	Machine operators	61	35.5
Junior High education	10	5.8	Binders/workers at the post-press	39	22.7
Middle school education	14	8.1	Supervisors / Production Managers	27	15.7
Technical education	23	13.4	Graphic designers	15	8.7
Senior High Secondary	62	36.0	Administrators	10	5.8
Diploma	20	11.6	Departmental / Sectional Heads	4	2.3
Bachelor’s Degree	35	20.3	Storekeepers	3	1.7
Post Graduate education	6	3.5	Client/Customer Service Personnel	2	1.2
**Work experience**			Estimators	1	0.6
		
Less than 1 year	28	16.3			
1–5	79	45.9			
6–10	38	22.1			
11-Above	26	15.1			

### Safety climate perceptions of workers in the Ghanaian printing industry

#### Safety climate in the printing industry

This study investigated the perceptions of print-production employees regarding safety climate on a 7-dimensional scale that covered: Safety Commitment & Communication, Safety Involvement & Training, Positive Safety Practices, Safety Competency, Safety Procedure, Accountability & Responsibility, and a Supportive Environment. The descriptive statistics for the levels of the safety climate index on each of the 7 dimensions and the individual items are given in Table 4.

**Table 4:** The general safety climate of the Ghanaian printing industry.

Safety commitment & communication	SD	D	N	A	SA	$\bar{x}$	*SD*
Sufficient feedback for proposals	19	64	29	34	24	2.88	1.26
Management’s interest in safety issues	22	97	15	22	16	2.49	1.15
Workers can openly discuss safety issues with the supervisor	29	97	13	26	7	2.33	1.05
Sufficient resource allocation	21	69	32	30	19	2.75	1.21
Respect for safe workers	19	97	18	19	13	2.46	1.09
Seeking for underlying factor to incidents rather than blaming people	8	87	43	25	6	2.61	0.92
Quick decision making when safety concern is raised	19	75	41	22	14	2.63	1.10
The company shows interest in my views on health and safety	16	85	29	25	16	2.65	1.13
Company’s interest in health and safety issues	25	84	29	17	16	2.50	1.15
Always provided with working equipment	17	75	40	28	9	2.63	1.05
Changes in procedures & environment are communicated effectively	18	100	15	23	16	2.53	1.14
Appropriate dissemination of safety and health information	20	58	39	37	12	2.78	1.14
Workers’ consultation on health and safety issues	11	64	35	50	11	2.92	1.09
Senior Level Management are involved with safety activities	14	73	40	30	13	2.74	1.09
Workers’ ability to influence safety performance at work	19	75	30	35	10	2.66	1.11
Manager’s understanding of operational issues that impact work safety	23	103	12	21	11	2.38	1.07
**Safety involvement & training**	**SD**	**D**	**N**	**A**	**SA**	$\bar{x}$	***SD***
							
Employees’ involvement in safety rules development/review	15	57	28	54	18	3.02	1.19
Related training during new procedure/equipment introduction	17	73	17	50	15	2.84	1.20
Strong encouragement to report unsafe condition	33	78	25	24	12	2.44	1.16
Workers’ consultation on training needs	16	44	33	58	17	3.10	1.18
Intervals for training to update knowledge	15	45	29	51	31	3.22	1.26
Company encourages suggestion on improving health & safety	15	88	24	30	12	2.62	1.10
Company training provides adequate skills & experience in operations	18	59	30	43	18	2.90	1.21
Accident investigation aims at finding cause not blaming individuals	23	58	43	26	17	2.74	1.18
**Positive safety practices**	**SD**	**D**	**N**	**A**	**SA**	$\bar{x}$	***SD***
							
Availability of enough people to always get the job done safely	27	90	18	32	5	2.41	1.05
General motivation by work task	29	97	22	18	5	2.26	0.96
Work site often being safe	31	83	20	21	16	2.46	1.19
Work load often being reasonably balanced	22	87	19	29	15	2.58	1.17
Company stop working to address safety concerns	30	44	27	48	23	2.94	1.33
Receiving of appropriate feedback for performance	20	81	28	32	10	2.60	1.10
Regulatory requirement performance at work place	14	51	41	46	19	3.03	1.16
Supervisor’s control over safety rule violation	18	63	36	38	15	2.82	1.16
**Safety competency**	**SD**	**D**	**N**	**A**	**SA**	$\bar{x}$	***SD***
							
Clarity on one’s responsibility for health and safety	23	109	9	19	11	2.33	1.05
Understanding of safety risks associated with one’s work	23	107	15	16	10	2.32	1.01
Understanding safety procedures associated with one’s work	23	107	19	12	8	2.26	0.95
**Safety procedure**	**SD**	**D**	**N**	**A**	**SA**	$\bar{x}$	***SD***
							
Following procedures to ensure work done safety	31	94	22	19	4	2.24	0.96
Safety is the number one priority in mind when working	42	83	18	22	6	2.22	1.07
Safety results reflect on how the job is now done	22	85	35	22	5	2.43	0.97
Safety procedures are written in unambiguous language for users	20	44	39	46	15	2.95	1.19
**Accountability & responsibility**	**SD**	**D**	**N**	**A**	**SA**	$\bar{x}$	***SD***
							
Workmate’s strong reaction against rules being broken	18	70	36	33	14	2.74	1.14
The written safety rules and instructions are easy for people to understand & implement	18	57	32	47	17	2.93	1.20
Co-workers often give tips to each other on how to work safely	11	106	18	25	10	2.51	1.02
**Supportive environment**	**SD**	**D**	**N**	**A**	**SA**	$\bar{x}$	***SD***
							
In my company, safety considerations are equally important as production	20	80	26	32	12	2.62	1.13
The safety rules always describe the safest way of working	19	66	35	42	9	2.74	1.11
Safety information is always brought to my attention by my line manager/supervisor	18	80	25	36	12	2.67	1.13

Strongly Disagree (SD) Disagree (D) Neutral (N) Agree (A)

Strongly Agree (SA) Mean ($\bar{x}$) Standard Deviation (SD)

In the study, the mean value of each dimension ranged from 1 to 5, so results below 3.0 translate as a negative or weak safety climate. Overall, the mean value of workers’ safety climate perception indices on all 7 dimensions (see Table 5) ranges from $\bar{x}$2.29 to $\bar{x}$2.82 with a total mean score of $\bar{x}$2.5844 (see Table 6). This value falls between “strongly disagree” and ‘‘disagree”. This shows a weak safety climate in the Ghanaian printing industry and may reflect the safety climate status of other sectors of the manufacturing industry in Ghana.

**Table 5:** Summary of dimensions mean score.

Safety dimensions	Mean	Std. Dev.	Variance	Cronbach’s Alpha (reliability)
Safety Commitment & Communication	2.59	0.76	.037	.90
Safety Involvement & Training	2.82	0.85	.070	.84
Positive Safety Practices	2.63	0.77	.089	.76
Safety Competency	2.29	0.91	.004	.86
Safety Procedure	2.42	0.83	.147	.74
Accountability & Responsibility	2.72	0.83	.075	.43
Supportive Environment	2.67	0.97	.004	.79

**Table 6:** Overall mean score.

Safety Climate		
N	Valid	172
	Missing	0
Mean		**2.5844**
Std. Deviation		.70336
Variance		.039
Cronbach’s Alpha (reliability)		.88

Further in-depth analysis of the individual dimensions provides a clearer insight into the workers’ perceptions of the safety climate of the printing industry in Ghana. The Safety Commitment and Communication dimension comprise 16 variables (see Table 3) that explain workers’ perceptions of the degree to which their managers and supervisors value and support a safe work environment and commit to workers’ safety.

The total mean score (see Table 6) of $\bar{x}$2.5844 shows an unfavourable perceived safety climate within the printing industry. This suggests that workers perceive management attitudes and actions as not positive about contributing to a healthy safety climate. Evidence such as lack of management’s understanding of operational issues that impact worker safety, lack of management’s interest in safety issues, and workers’ inability to discuss safety issues with supervisors, are indicators of the lack of management’s commitment to safety in the companies. Providing inadequate “safety competency” and “safety procedure” (see Table 5) for employees, with a factor mean score of $\bar{x}$2.29 and $\bar{x}$2.42 respectively also indicates a poor safety climate in the companies.

Though Safety Involvement & Training dimension scored, $\bar{x}$2.82 is close to the positive mean of $\bar{x}$3.0, the mean score of $\bar{x}$2.82 still shows a fairly low safety climate within the industry. Since safety climate studies help to assess an overall sense of shared beliefs, values and traditions around workplace safety within the larger framework of organizational systems, the outcome of this study shows a weak and negative safety climate within the Ghanaian printing industry. This position is supported by employee safety-related research, that shows a high incidence of injuries, especially where employees’ perceptions of a company’s safety climate are low and low when perception is high [53–55].

It is imperative, therefore, that management provides an acute sense of leadership in safety matters to point an organisation’s behaviour in the right direction. If safety climate is an effective predictor of safety-related behaviours, an organisation’s safety performance and safety outcomes [including injuries and accidents] [56–58] and antecedents of workplace accidents, then this study has revealed the looming threat to human life and safety within the printing industry. Management must, therefore, show a commitment to occupational health and safety programmes to prevent future injuries and fatalities.

## Demographic characteristics and safety climate

As already indicated, it is important to investigate the potential relationship between moderating variables and safety climate. In this section, we report on potential moderating factors on demographic variables–age, gender, education, experience and size of organisation–concerning perceived safety climate.

### Gender and perception of workplace safety

***Hypothesis 1**. There is a relationship between gender and individual perception of the workplace safety climate*.

In testing the hypothesis on gender and safety climate, an independent-sample t-test conducted to compare male safety climate scores with female safety climate scores found a significant difference in the scores for males (M = 2.6, SD = 0.70) and females (M = 2.3, SD = 0.65) safety climate perception; *t* (170) = 2.42, *p* = 0.16. These results suggest gender influences safety climate perception. Specifically, our results suggest females perceive the climate as more negative compared to males, even though they both recorded a negative perception score on the Safety Climate Scale.

### Work experience and perception of workplace safety

***Hypothesis 2:** There is a relationship between years of work experience and perception of the workplace environment*

On work experience, the data showed a statistically significant difference in the score of less experienced workers (M = 2.47, SD = 0.633) and more experienced workers (M = 2.75, SD = 0.79) safety climate perception; *t* (170) = -2.58, *p* = 0.10). This shows that experienced employees (more than 5 years of working experience) perceive the work environment as safer compared with less experienced employees (not more than 5 years of working experience). This confirms Gyekye and Salminen’s [59] work that found that workers less experienced were the “least enthusiastic” about the safety climate in their organisation. This observed relationship between years of workplace experience and safety perception could be because of the concept of familiarity and perception of hazards [59]. That is when people become familiar with several aspects of their environment, their perception level of hazards turns to diminish [60] and known risks are more acceptable, as though they do not exist.

### Age and perception of workplace safety

*Hypothesis 3: There is a relationship between age and individual perception of the workplace climate*.

The result shows a statistically significant difference, *t* (170) = 2.754, *p* = .007, between the perception of younger employees 35-year-olds and below, (M = 2.6851, .66622), and older employees 36-year-olds and above (M = 2.3757, .737882). This result suggests that older employees aged 36 and above have a lower perception of the safety climate compared to those aged 19 to 35 years, considered younger employees. This means older employees are less likely to follow through with safety commitment, safety priority and risk non-acceptance. While previous studies in age and safety perceptions reveal mixed results, our findings are consistent with the findings of Glendon and Litherland [40], which found significant relationships between age and safety climate perceptions in the road construction industry.

### Educational attainment and perception of workplace safety

*Hypothesis 4. There is a relationship between educational attainment and individual perception of the workplace climate*.

Out of the subgroup found with low educational attainment (basic, secondary, and middle school graduates) the independent sample t-test indicated the self-reported safety climate perception was more negative than the self-reported safety climate perception of the highly educated, (M = 2.84, SD = 0.83026), at a statistically significant difference of *t* (169) = -3.713, *p* = 0.001).

The results suggest that the perception of a safe work climate increases with the level of education. The outcome proves that the safety climate perception of employees with basic/secondary education is very low (negative) compared with those with tertiary education, although they both scored negatively on SCSMI. This denotes that employees with a low level of education perceive safety at their workplaces as more negative than the employees with a high level of education. This position contradicts other research findings that measure the level of education and safety climate. An explanation for our findings could be because employees with tertiary education usually work in administration in press houses where the risks are comparatively minimal than those who work at the press and post-press sections of the organisation, they are likely to perceive their safety climate as safer.

### Organisation size and perception of workplace safety

*Hypothesis 5: There is a relationship between company size and employee perception of the safety climate*.

The subgroup analysis for the type of organisations by size—micro, small, medium and large—was assisted by ANOVA. In examining the subgroup differences, we found no significant subgroup differences. The homogeneity of variance test involved using Levene’s Test and found tenable with *f* (3,168) = 0.99, *p* = 0.398. Between-groups mean significance level (*p* = 0.407) which is above 0.05, shows the safety climate between groups has no statistically significant difference. Thus, the overall ANOVA was not statistically significant. We, therefore, suggest that the negative safety climate cuts across many organisations and this perception is consistent with all employees regardless of the size of the organisation.

## Conclusion

The results showed that all the socio-demographic characteristics had significant relationships with safety climate perception in the industry. These findings are consistent with the findings of Choudhry et al. [61] who reported that demographic factors such as age, sex, and level of work experience influence safety climate perception and consequently influence an employee’s safety behaviour; and by extension may play a significant role in risk-taking behaviour at the work place [62].

Indeed, if the outcome of the organisation’s safety climate study can be an effective indicator of the "safety health state" of the company, and a predictor of workplace injuries, then the conclusion we draw from this study is that the current climate of the Ghanaian printing industry is not safe. Workers perceived management attitudes and actions as unfavourable since safety issues were not a priority for management. The results from measuring the dimensions: safety commitment and communication, safety involvement and training, positive safety practices, safety competency, safety procedures, accountability and responsibility and supportive environment, all show an unhealthy or a negative safety climate in the printing industry. Hence, one should expect the incidence of high-level occupational accidents [63], accident under-reporting [64] and the incidence of increased risk-taking behaviours of the workers within the printing industry of Ghana.

To improve the safety climate, management and other stakeholders within the printing industry must change their attitude towards safety. They should commit to and communicate effectively to embrace safety practices and procedures. Management should avoid palliative actions, i.e. wait for safety incidents to happen in an organisation before implementing vigorous safety measures, rather they should adopt preventive actions, and be more accountable and responsible to minimize or eliminate the effects of a poor safety climate.
